# A Review of Last Decade Developments on Epiretinal Membrane Pathogenesis

**Published:** 2020-03-20

**Authors:** Eleni Tsotridou, Eleftherios Loukovitis, Konstantinos Zapsalis, Iro Pentara, Solon Asteriadis, Paris Tranos, Zachos Zachariadis, George Anogeianakis

**Affiliations:** 1 Ophthalmica Eye Institute, Thessaloniki, Greece.; 2 Faculty of Medicine, Aristotle University of Thessaloniki, Thessaloniki, Greece.; 3 Department of Ophthalmology, 424 General Military Hospital, Thessaloniki, Greece.; 4 Association for Training in Biomedical Technology, Thessaloniki, Greece.

**Keywords:** Epiretinal Membrane, ERM, Pathogenesis, Idiopathic, Secondary, Cell types, Trophic Factors, Transcription Factors

## Abstract

Epiretinal membrane (ERM) is a pathologic tissue that develops at the vitreoretinal interface. ERM is responsible for pathological changes of vision with varying degrees of clinical significance. It is either idiopathic or secondary to a wide variety of diseases such as proliferative diabetic retinopathy (PDR) and proliferative vitreoretinopathy (PVR). A great variation in the prevalence of idiopathic ERM among different ethnic groups proposed that genetic and lifestyle factors may play a role in ERM occurrence. Histopathological studies demonstrate that various cell types including retinal pigment epithelium (RPE) cells, fibrocytes, fibrous astrocytes, myofibroblast-like cells, glial cells, endothelial cells (ECs) and macrophages, as well as trophic and transcription factors, including transforming growth factor (TGF), vascular endothelial growth factor (VEGF), platelet-derived growth factor (PDGF) etc., are directly or indirectly involved in the pathogenesis of idiopathic or secondary ERMs. These processes are driven (on the last count) by more than 50 genes, such as Tumor Necrosis Factor (TNF), CCL2 (chemokine (C-C motif) ligand )), Metastasis Associated Lung Adenocarcinoma Transcript 1 )MALAT1(, transforming growth factor (TGF)-β1, TGF-β2, Interleukin-6 (IL-6), IL-10, VEGF and glial fibrillary acidic protein (GFAP), some of which have been studied more intensely than others. The present paper tried to summarize, highlight and cross-correlate the major findings made in the last decade on the function of these genes and their association with different types of cells, genes and gene expression products in the ERM formation.

## INTRODUCTION

Epiretinal membrane (ERM) is a pathologic layer, located at the vitreoretinal interface, which grows on the inner surface of the retina [1, 2]. It is mainly localized on the central retina or the surface of macula [3]. ERM was first described by Iwanoff in 1865 [4-8]. It has been designated by many names, such as primary retinal folds, wrinkling of the inner retinal surface, silent central retinal vein obstruction, preretinal macular fibrosis (PMF) or gliosis, or macular pucker and cellophane maculopathy [3]. 

ERMs can be either idiopathic or secondary [9-13]. In idiopathic or primary ERM, cell proliferation appears following posterior vitreous detachment (PVD) [9], in up to 95% of the cases [14] and a break in Internal Limiting Membrane (ILM). Furthermore, two types of idiopathic ERM have been introduced. Type I refers to cases where vitreous collagens intervene between ILM and ERM, while the second type describes those where cell proliferation takes place directly on the ILM surface with only a sparse or even no collagen containing layer forming between them [15]. 

**Table 1:** Structure of the Present Review

Structure of the Review
Abstract
Introduction
Method
General aspects of ERM pathogenesis
Clinical Signs
Epidemiology and Risk Factors
The Genetic and Proteomic Basis of ERM Pathogenesis
Cells Involved in ERM Pathogenesis
Genes Involved in ERM Pathogenesis
Collagens and ERM
Changes of Cytokines and Growth Factors in ERM Formation
The TGF-β Family
NGF
VEGF
IGF
The PDGF receptor
GDNF
Neurotrophin receptors
Changes of Other Proteins Specific to Idiopathic ERM
Changes of Proteins Occur in Both Idiopathic and Secondary ERMs
Inflammation Molecules in Idiopathic and Secondary ERM
Conclusions - Closing Comments
References

Abbreviations: ERM: epiretinal membrane; NGF: nerve growth factor; VEGF: vascular endothelial growth factor; IGF: insulin-like growth factor; PDGF: platelet-derived growth factor; GDNF: glial cell-line-derived growth factor

Secondary ERMs are the result of an already existing ocular pathology, including vitreoretinal vascular disorders. Common causes include PVR, PDR, hypertensive retinopathy as well as diseases like intraocular inflammation (e.g., uveitis), occlusion of a central or branch retinal vein, retinal detachment (RD) and ocular trauma [9-12]. Secondary ERMs are also attributed to retinal tears, retinal photocoagulation, cryotherapy, retinal vasculitis, vitreal hemorrhages and retinal surgical procedures [9, 10, 16]. ERMs therefore, can be vascularized (e.g., in eyes with proliferative retinopathies), or avascular [10]. However, secondary ERMs are less aggressive and seldom cause traction RD as do primary ERMs [17]. 

Structurally, ERM includes an inner and an outer extracellular matrix (ECM) layer. The first one includes one or multiple cell layers while the second one contains randomly oriented extracellular fibrils, in most cases [15, 18, 19]. The epiretinal cells, of either retinal or extraretinal origin may be glial cells (Müller cells and astrocytes), RPE, fibrocytes, myofibroblasts, fibroastrocytes, laminocytes, hyalocytes, macrophages or fibroblasts [6, 12, 16, 18]. In its outer layer, the ECM includes extracellular fibrils, fragments of ILM, residual native vitreous fibrils in cases like vitreoschisis or partial PVD [19]. It also contains proteins, e.g., fibronectin (FN), vitronectin (VTN) or collagen [6, 16, 18]. 

Nonangiogenic fibroglial tissue characterizes idiopathic ERMs, in contrast to PDR ERMs which are mainly characterized by neovascularization [20, 21]. ERM is the most severe stage of PDR, because of retinal traction or tractional RD secondary to ERM contraction [22]. However, both idiopathic and secondary ERM formation have poorly understood molecular mechanisms [20], although, glial cell proliferation which follows PDR [20, 23] is suspected to have a vital role in ERM formation. 

Fibrocellular proliferation of ILM and cellular contraction are also ERM features. PVD can injure the ILM, allowing movement of glial cells to the retinal surface and favorable conditions for fibrocellular proliferation, between the vitreous and the retina. This might be provided by an incomplete PVD as well [4-7]. 

The present paper tried to summarize (Tables 1, 6), highlight and cross-correlate (Tables 2, 3, 4, 5) the major findings made in the last decade on the function of these genes and their association with different types of cells, genes and gene expression products involved in ERM pathogenesis.

Based on the review literature, this article focuses and attempts to summarize, highlight and cross-correlate the major findings about the role of cells, genes and gene expression products implicated in ERM formation, idiopathic and secondary.

## METHOD

This was a literature research on PubMed and the Scholar databases for articles related to the pathogenesis of ERM, idiopathic or secondary. “Epiretinal membrane”, “genes”, “genetic associations”, “proteins”, “growth factors” and “pathogenesis” are the keyword combinations used. The research represents a critical appraisal focused on articles dated from January 2008 until December 2019. A total of 60 articles were identified, and the analysis of their references revealed an additional 78 articles related to ERM pathogenesis, which were also reviewed. 

**General aspects of ERM pathogenesis**

Migration of retinal-tissue-derived glial cells from the optic disc together with microdefects in the ILM (retinal pits or holes), which occur during PVD and move towards the surface of the retina may contribute to the pathogenesis of ERM [3, 15, 24, 25]. Other theories suggest that as an incomplete PVD allows the membrane proliferation between the vitreous and the retina, the pathogenesis of ERM is associated with the growth and fibrous metaplasia of the vitreous cells [9, 14, 16]. The retinal break provides suitable conditions for the migration of the RPE cells to the vitreous cavity and migration to the retinal surface, forming the ERM in the case of rhegmatogenous RD [24, 26]. Thus, PVD seems to be a crucial pathogenic factor in idiopathic ERM formation as it is encountered in 70% of patients at the earlier stages of disease [19]. Modifications of the ERM macromolecules may increase membrane stiffness and provide suitable conditions for the fibrotic process. Glial cells, fibroblasts, hyalocytes, along with cytokines and growth factors present in the vitreous fluid (VF) may contribute to ERM pathogenesis. Although there is a debate about which types of cells are responsible for idiopathic ERMs and the pathogenetic ways by which they migrate to the retinal surface [15].

**Clinical Signs**

ERMs impact negatively the quality of life leading to significant visual impairment [27, 28]. They are characterized by several pathological changes occurring in the vitreoretinal junction differently expressed with various clinical signs [24, 25, 29, 30]. Clinically, ERMs appear on ophthalmoscopic examination as a translucent, transparent, or pigmented membrane, or like a glistening light reflection when the ERM is thin [3, 11, 19]. 

Although, the early form of thin and translucent ERM is usually non-symptomatic or may provoke minor changes in visual acuity, which is seldom below 20/200, the more severe forms characterized by a semi translucent, thick, and contractile membrane can cause significant loss of visual acuity and a number of visual symptoms [3, 11, 19, 31, 32]. ERMs may be peripheral or macular. Macular ERMs are more visually disturbing [6, 33, 34]. They may involve macular and/or perimacular regions causing a reduction in visual acuity, micropsia, metamorphopsia, retinal wrinkling, distortion, blurred vision and occasionally monocular diplopia [12, 14, 19, 31, 35, 36]. However, absolute scotomas are rare [3]. ERMs may also cause vitreoretinal tractions or even tractional RD [12] as they often exert traction on the underlying retina, reducing visual acuity when they result in tractional detachment of the macula [3, 10]. Symptoms may differ individually depending on the duration and severity of disease [12]. 

**Epidemiology and Risk Factors**

Despite extensive observations about ERM, its pathogenesis and pathophysiology are still poorly understood [9, 37]. 

The most consistently identified risk factors for ERM are age and PVD [13, 19, 38], with PVD encountered in 70% of patients at the earlier stages of disease [19]. Occasionally, ERMs develop in children and young adults [3], but prevalence of ERM increases with age with most ERMs occurring in individuals older than 50 years [3, 14, 28, 38]. It is estimated that the incidence approaches 20% of the total population by the age of 70 years [24, 25], while the prevalence of macular ERMs is estimated to be 2% when patients are younger than 60 years, 12% in patients over 70 years [6, 33] and 22.5% in those aged 80 years or more [19, 31, 39]. Other population studies indicate that overall ERM prevalence is 7% to 11.8%, while the 5-year incidence is 5.3% [1, 6, 7, 33, 37, 40, 41]. 

ERM prevalence does not differ significantly between men and women [19, 31, 39]. On the other hand, it shows substantial racial and ethnic variation [28]. Epidemiologic studies, among different ethnic groups, report great discrepancies in the prevalence of idiopathic ERM, ranging from 1.02% to 28.9%. ERM is more prevalent in the United States (USA) and Australia compared to Asian countries, like China, Japan and Singapore, pointing to genetics and lifestyle [18, 19] as predisposing factors. Interestingly, these studies indicated no important differences of the prevalence of PMF, a severe form of idiopathic ERM, which should be treated with surgical intervention [19]. 

There are almost 30 million people in the USA having ERM in at least one of their eyes. Moreover, the prevalence seems to be lower in Asia, markedly lower in China [11, 28] but higher in Asian Malays, compared with Caucasians [38], but it is similar between Japanese and Caucasians. Finally, the prevalence of ERM among those of Southern European origin appears to be twice that in those of Northern European origin, while a high prevalence is also reported for the U.S. Latinos [11, 28]. 

The role of race and ethnicity in the prevalence of ERM has remained unclear [13, 14, 40]. However, they may relate to the different study methods used including sampling, photography etc., or the definition of ERM used, especially when the clinical signs are not already obvious [28]. Ethnic variations may also relate to different genetic predisposition, clinical factors, comorbidities (e.g., diabetes), previous cataract surgery and lifestyle factors (e.g., smoking). Exposure to unknown risk factors may also be involved. Nonetheless, as mentioned above, increasing age is the most common risk factor for any type of ERM [27]. 

Increased prevalence of idiopathic ERM accompanies diabetes, hypercholesterolemia and vascular narrowing or occlusion. These associations suggest a strong role for metabolic factors [18, 19] in the development of ERM. In addition, cardiovascular risk factors appear to be associated, albeit inconsistently, with idiopathic ERMs [28]. However, this may be a manifestation of metabolic factors mentioned, given that they are causative factors of cardiovascular disease. Finally, ERM could be associated with refractive errors, although relevant data are inconsistent; a number of studies indicated increased prevalence of idiopathic ERM in hypermetropic eyes, while others implicated myopic eyes [19]. 

**The Genetic and Proteomic Basis of ERM Pathogenesis**

Despite our incomplete knowledge of the pathogenesis of ERM [9], gene expression profiles carried under different conditions [17, 42] have identified several genes as crucial for ERM formation. Modern imaging in coordination with immunocytochemistry and proteomic techniques, improved our knowledge of the pathogenesis of idiopathic and secondary ERMs [15]. Apparently, not one or just a few growth factors influence the pathogenesis of ERMs [43]. Indeed, ERM appears to be mainly an avascular fibrocellular proliferation, given that a number of ECM proteins and cells like RPE and glial cells are components of ERM [9, 44]. 

**Cells Involved in ERM Pathogenesis**

ERMs are characterized by cellular migration and proliferation on the inner retinal surface [37], ECM formation and tissue contraction [29]. Histopathological studies indicate that RPE cells, glial cells, macrophages, ECs, fibrocytes, fibrous astrocytes and myofibroblast-like cells, as well as trophic and transcription factors contribute to ERM formation [1, 10, 24, 25, 29, 35, 45-51]. However, the stimuli causing epiretinal and intravitreal cellular proliferation and ERM formation are unknown. Imprecise morphologic criteria for cell-type identification, secretion of extracellular material and morphologic changes that occur in cells during proliferation and maturation of ERM contribute to the uncertainties about which cell types are involved in ERM pathogenesis [10]. 

RPE cells is apparently the major cell type contributing to ERMs and intravitreal membranes in eyes with rhegmatogenous RD, which is complicated by massive periretinal proliferation [10]. RPE cells are hexagonal quiescent cells that neither proliferate nor migrate under physiological condition [52, 53]. However, there are several cell types (e.g., glial cells) which contribute to the fibrotic reaction in RD [54, 55], by way of protecting the retina through the formation of a scar-like thin layer [20, 23, 29, 47, 48, 56, 57]. 

Other cell types whose origin is not fully elucidated, especially in diabetic ERM, are fibroblasts [57, 58]. These cells synthesize the ECM, construct the structural framework of the membrane, are capable of producing collagen and, as a result, can cause tractional RD [57]. Myofibroblasts, possibly of hyalocyte, Müller cell, or RPE cell origin predominate in late idiopathic ERMs and have a crucial role in idiopathic ERM pathogenesis since they secrete contractile proteins, induce intracellular contraction and deposit collagen [14]. 

Müller cells are other type of cells with central role in ERM contraction due to their ability to express a-smooth muscle actin (a-SMA), which is involved in membrane contraction and produce various collagens [1, 18, 20, 23, 59, 60]. Idiopathic ERM formation grows as Müller cells proliferate, migrate and transdifferentiate. Müller glial cells (MGCs) contribute to the formation of ERM, by acquiring migratory ability and exhibiting a fibroblast-like phenotype, stretching the retina and causing RD and vitreous hemorrhage. They are also involved in the formation of diabetic ERM, which is associated with reactive gliosis, fibrosis and migration [57, 61]. Laminocytes are other cell types with fundamental involvement in the pathology of idiopathic ERMs. These are dispersed in reduced numbers in eyes containing a PVD [29]. Additionally, ERM formation and contraction are enhanced by the activation of hyalocytes that are placed on the cortical vitreous remnants. This process is enhanced by different growth factors contributing to the cellular proliferation and myofibroblast differentiation [10, 29] (Table 2). During the formation of idiopathic ERM, TGF-β is produced and activated by both hyalocytes and retinal Müller cells [18, 68-70].

**Table 2:** Some Cell Types in ERM Pathogenesis

Cell Type	Role
Astrocytes	Idiopathic ERM [47]
Endothelial cells	ERM formation [47, 62]
Fibroblasts	Idiopathic [63] and secondary (PDR) ERM [57, 58, 63]
Fibrocytes	ERM formation [29, 35, 47]
Glial cells	Idiopathic and secondary (PVR) [64, 65] (PDR) [29, 50, 57, 61, 64] ERM
Hyalocytes	Idiopathic and secondary (PVR and PDR) ERM [9, 47, 66, 67]
Laminocytes	Idiopathic ERM [29]
Macrophages	Idiopathic ERM [10] and secondary (PVR) ERM [64]
Müller cells	Idiopathic [14, 18, 57, 61, 63, 69, 70] and secondary ERM [63]
Myofibroblast-like cells	Idiopathic [14, 47] and (PVR and PDR) secondary ERM [9, 47, 66, 67]
RPE cells	Idiopathic and (PVR and PDR) secondary ERM [9, 29, 47, 64, 66, 67]

Abbreviations: ERM: epiretinal membrane; PDR: proliferative diabetic retinopathy; PVR: proliferative vitreoretinopathy; RPE: retinal pigment epithelium

TGF-β contributes to ERM contraction by upregulating the expression of a-SMA in the epiretinal cells. Non-expressing a-SMA retinal Müller cells apparently produce collagens that form the ECM network in idiopathic ERM, while it is the retinal Müller cells that express a-SMA responsible for the membrane contraction [18]. The role of many other cell types, like macrophages, in the pathogenesis of idiopathic ERMs has yet to be determined [10].

**Genes Involved in ERM Pathogenesis**

Several changes take place in gene expression during the onset as well as later in progress of various diseases [17, 71]. Genes with a crucial role in any particular disease can be identified through comparison of different gene expression profiles [17, 42]. Therefore, the analysis of gene expression can enhance the understanding of ERM formation. 

The expression of genes in idiopathic or secondary ERM has been extensively studied [47]. There are at least 52 genes upregulated in either PVR or secondary ERMs. The expression of 29 genes is increased in secondary ERMs as the expression of 23 genes in PVR-ERMs. Four genes in particular, ZNF713, FN1, MALAT1 and PARP8, are highly upregulated in ERMs. Genes that contribute to proliferation and cell adhesion are highly expressed in PVR-ERMs, while in the rest of secondary ERMs, genes which are implicated in metabolism, ribosomes and signaling are slightly elevated [17]. Ten cell adhesion genes expressed in the PVR-ERMs are *COL1A1, COL1A2, COL3A1, POSTN, THBS1, LGALS1, SPARC, FN1, TIMP3 and DCN. *These findings are supported by morphological studies that enhance the positive correlation between the disease process with the amount of ECM in ERMs, especially in PVR-ERMs [17, 72]. Genes like MALAT1, STAT3, CD320 and SERPINE1 which are implicated in proliferation, are also upregulated in PVR-ERMs. In addition*, several other genes and proteins, *among them 60 genes not detected in PVR-ERM, *may also be involved* [17]. 

Of genes mentioned above, MALAT1 is associated with ERM formation, with a crucial role in the progression of secondary, especially PVR, ERM. This gene is a large, non-coding RNA, highly conserved among mammals, which appears to be the most actively expressed PVR-ERM gene. It is involved in the expression of genes associated with metastasis and motility at the level of transcription and/or post-transcription regulation [17, 73]. As PVR-ERMs do not synthesize MALAT1, it is suggested that cell migration of PVR-ERMs on the retina may trigger the expression of MALAT1 in ERM [17]. 

The expression of genes like RELA (v-rel avian reticuloendotheliosis viral oncogene homolog A), tenascin C (TNC), glial fibrillary acidic protein (GFAP) and other cytokine-encoding genes such as TGF-β2, IL-6, VEGFA and CXCL1 (chemokine C-X-C motif ligand 1) is intensified irrespective of sex, especially in idiopathic ERM eyes [47]. RELA is a component of the nuclear transcription factor kappa B (NF-κB). Hypoxia, infections by viruses and bacteria and proinflammatory cytokines such as IL1β and tumor necrosis factor alpha (TNF-a), apparently activate NF-κB [47, 74, 75]. It is suggested that a consequence of old age is the abundant representation of RELA in ECs and glial cells, which leads to upregulation of NF-κB, stimulation of the proliferation of glial cells [50, 62, 76] and IL-8, resulting in ERM formation [47, 62]. Numerous NF-κB target genes, among them proinflammatory cytokines, like IL1β and IL-6 and TNF-a (which are vital contributors to the formation of ERMs and the pathological process of DR) are potentially upregulated by activated glial cells [20, 66]. Indeed, patients with PDR ERMs have significantly higher NF-κB mRNA expression levels than those in idiopathic ERMs [50, 51].

Another gene that possibly plays an important role in ERM formation is the PPM1D gene. This gene encodes Wild-type p53-induced phosphatase 1 (Wip1), which has a key role in stress signaling [50, 77], inflammation, cell survival, cell cycle progression and proliferation [50, 78]. In addition, there are several reports about the relation between ERMs of PDR and the expression of Wip1. Although Wip1 may be associated with ERM formation, little is known about this association [50]. 

SP1 mRNA (specificity protein 1) is also highly represented in PDR-ERM. SP1 regulates expression of genes that encode angiogenesis-related factors and has a vital role in PDR angiogenesis [79]. SP1 regulates the expression of several genes, such as TGF-β, VEGF, fibrogenic cytokine and many matrix genes, while the SP1 coded protein is co-localized mainly with VEGF. These cytokines have been detected in the VF and PDR-ERMs [79, 80] (Table 3). 

Genes associated with wound healing and angiogenesis along with the pro-inflammatory genes, *TGF-β2, IL-6*, *RELA*, *GFAP, VEGFA*, *TNC* and *CXCL1* are slightly more expressed in eyes with idiopathic ERM [47], while there is a positive intraocular fibrosis and is upregulated in eyes with PVR and PDR ERM [9, 20, 29, 47, 57, 66, 67] and induce the transformation of RPE cells, hyalocytes and myofibroblastic cells [9, 29, 47, 57, 67].

**Table 3:** Genes, the Proteins They Encode and Their Role in ERM Pathogenesis

GENES	PROTEINS ENCODED	ROLE
CD320 (CD320 Molecule)	CD320 antigen	related to proliferation (PVR-ERM) [17]
*COL1A1 *	Collagen, type I, alpha 1	adhesion-related gene (PVR-ERM) [17]
*COL1A2*	Collagen, type I, alpha 2	adhesion-related gene (PVR-ERM) [17]
*COL3A1 *	Collagen type III, alpha-1	adhesion-related gene (PVR-ERM) [17]
CXCL1 (C-X-C Motif Chemokine Ligand 1)	C-X-C Motif Chemokine Ligand 1	cytokine-encoding gene (idiopathic ERM) [47]
*DCN (*DeCoRin)	Decorin	adhesion-related gene (PVR-ERM) [17]
FN1 (FibroNectin-1)	Fibronectin	adhesion-related gene (PVR-ERMs and secondary ERM) [17]
GFAP (Glial Fibrillary Acidic Protein)	Glial fibrillary acidic protein	pro-inflammatory genes (idiopathic ERM) [47, 81]
IL-6 (InterLeukin-6)	Interleukin-6	cytokine-encoding gene (idiopathic ERM) [47]
*LGALS1 (*GALectin 1)	Galectin-1	adhesion-related gene (PVR-ERM) [17]
MALAT1 (RNA gene) (Metastasis Associated Lung Adenocarcinoma Transcript 1)	non-coding RNA	related to proliferation/ metastasis/ regulates processing pre-mRNAs in mammalian cells and cell motility (PVR-ERMs and secondary ERM) [17]
PARP8 (Poly(ADP-Ribose) Polymerase 8)	Poly [ADP-Ribose] Polymerase 8	(PVR-ERMs and secondary ERM) [17]
*POSTN (*PeriOSTiN)	Periostin	adhesion-related gene (PVR-ERM) [17]
PPM1D (Protein Phosphatase, Mg2+/Mn2+ Dependent 1D)	Wild-type p53-induced phosphatase 1 (Wip1)	active in stress signaling, inflammation, cell survival, cell cycle progression and proliferation (PDR-ERM) [50, 77]
RELA (v-REL avian reticuloendotheliosis viral oncogene homolog A)	nuclear factor NF-kappa-B p65 subunit	regulation of cellular signal transmission, cell migration and apoptosis, and tumor growth and progression (idiopathic ERM) [47]
SERPINE1 (SERPIN Family E Member 1)	Serpin family E member 1	related to proliferation (PVR-ERM) [17]
SP1 (Sp1 Transcription Factor)	Sp1 transcription factor	angiogenesis (PDR-ERM) [79]
*SPARC *(Secreted Protein Acidic and Cysteine Rich)	Osteonectin	adhesion-related gene (PVR-ERM) [17]
STAT3 (Signal Transducer and Activator of Transcription 3)	Signal transducer and activator of transcription 3	related to proliferation (PVR-ERM) [17]
TGFβ2 (Transforming Growth Factor Beta 2)	Transforming growth factor beta 2	cytokine-encoding gene (idiopathic ERM) [47]
*THBS1 (*THromBoSpondin-1)	Thrombospondin-1	adhesion-related gene (PVR-ERM) [17]
*TIMP3 (*TIMP Metallopeptidase Inhibitor 3)	Metalloproteinase inhibitor 3	adhesion-related gene (PVR-ERM) [17]
TNC (Tenascin C)	Tenascin C	pro-inflammatory gene (idiopathic ERM) [47, 81]
VEGFA (Vascular Endothelial Growth Factor A)	Vascular endothelial growth factor A	cytokine-encoding gene idiopathic ERM) [47]
ZNF713 (Zinc Finger Protein 713)	Zinc finger protein 713	transcriptional regulation (PVR-ERMs and secondary ERM)[17]

Abbreviations: PVR: proliferative vitreoretinopathy; ERM: epiretinal membrane; COL1A1: collagen, type I, alpha 1; COL1A2: collagen, type I, alpha 2; COL3A1: collagen type III, alpha-1; CXCL1: C-X-C Motif Chemokine Ligand 1; DCN: decorin; FN1: fibronectin-1; GFAP: glial fibrillary acidic protein; IL-6: interleukin-6; LGALS1: galectin 1; MALAT1: metastasis associated lung adenocarcinoma transcript 1; PARP8: poly (ADP-Ribose) polymerase 8; POSTN: periostin; PPM1D: protein phosphatase, Mg2+/Mn2+ dependent 1D; Wip1: wild-type p53-induced phosphatase 1; PDR: proliferative diabetic retinopathy; RELA: v-REL avian reticuloendotheliosis viral oncogene homolog A; SERPINE1: SERPIN family E member 1; SP1: Sp1 transcription factor; SPARC: secreted protein acidic and cysteine rich; STAT3: signal transducer and activator of transcription 3; TGFβ2: transforming growth factor beta 2; THBS1: thrombospondin-1; TIMP3: TIMP metallopeptidase inhibitor 3; TNC: tenascin C; VEGFA: vascular endothelial growth factor A; ZNF713: zinc finger protein 713

Glial cells like astrocytes or MGCs, which express GFAP, are associated with idiopathic ERM. Retinal glial cells produce VEGF [25, 47], whereas idiopathic ERMs are characterized by the association in gene expression levels between VEGFA and GFAP. TNC has a vital role in the sprouting of endothelial cells (ECs) cells during angiogenesis and also in several processes like inflammation and wound healing [47, 81]. Therefore, TGF-β2 and the factors, CXCL1, IL-6 and NF-κB, are potential stimuli for GFAP positive cells that contribute to the process of fibrosis, while VEGFA and TNC may enhance idiopathic ERM formation [47].

Finally, the expression of several genes that encode cytokines in ERM eyes is enhanced, while it remains stable for other cytokine encoding genes. As mentioned above, genes like RELA, VEGFA, IL-6, TGF-β2, CXCL1, GFAP and TNC are significantly upregulated in the idiopathic ERM eyes, while ACTA1 and IL17A are not detected in idiopathic ERMs. In addition, the levels of CCL2 (chemokine (C-C motif) ligand 2), CCL5, CXCL10, TGF-β1, TNF, STAT3, RORC, TBX21, IFNG and POSTN does not change in idiopathic ERM [47]*. *

**Collagens and ERM**

Given the increased collagen deposition and membrane contraction, the ERM progression is a fibrotic process. Collagen plays a crucial role in ERM construction and collagen types I-VI are important components of idiopathic ERM [18, 82, 83]. It also has an essential role in proliferation and migration. Moreover, collagens bind to the cellular membrane receptors and have a significant role in matrix remodeling, fibrosis and cell signaling [44]. 

On the basis of their collagenous fibrillar ECM, two major types of idiopathic ERM can be identified. One of them is characterized by high amounts of fibrils consisting of collagen type VI; the other by high amounts collagen types I and II [18, 83]. The role of collagen type VI seems to be vital because of its contribution to the proliferation, migration and fibroblasts to myofibroblasts transdifferentiation. Fibroblast transdifferentiation is a vital procedure for fibrosis, matrix remodeling and scar contraction in many fibrotic diseases including ERM [18, 44]. Type VI also contributes to a fine fibrillar network whose presence characterizes idiopathic ERM [18, 83-85]. 

Collagen types I, III and IV are freshly produced during idiopathic ERM formation. They promote the fibrotic process and contribute to the formation of hard collagen scaffold, where the myofibroblast precursor cells, when anchored there, become more susceptible to fibrogenic factors [19]. Collagen types III and IV, FN, and laminin are present in both early and late idiopathic ERMs [14] (Table 4).

Newly formed ERM collagens along with the density of the associated cells apparently enhance vitreoretinal adhesion. Collagens types I, III and V enhance mechanical functions, are major constituents of skin, muscles, blood vessels and fibrotic scar tissues, and can affect the mechanical properties of ERM. However, collagens types I, III and V do not appear in the normal vitreoretinal interface. Recently formed collagens not only provide strong adhesion to various tissues, but also influence the rigidity and digestibility of ERM [44]. 

**Table 4:** Different Collagen Types and Their Role in ERM

Collagen Type	Role
Collagen type I	Promotes the fibrotic process and contributes to the formation of the hard collagen scaffold, where the myofibroblast precursor cells, when anchored there, become more susceptible to fibrogenic factors [19]. Also, it affects the mechanical properties of ERM. Although it enhances adhesion to various tissues, recently formed collagens may influence the rigidity and digestibility of ERM. Important component of idiopathic ERM [18, 44].
Collagen type II	Proliferation and migration. It binds to cellular membrane receptors, while it has a significant role in matrix remodeling, fibrosis mediation and cell signaling. Important component of idiopathic ERM [18, 44].
Collagen type III	Promotes the fibrotic process and contributes to the formation of the hard collagen scaffold, where the myofibroblast precursor cells, when anchored there, become more susceptible to fibrogenic factors [19]. Also, it affects the mechanical properties of ERM. In addition to enhancing adhesion to various tissues, recently formed collagens may influence the rigidity and digestibility of ERM. Important component of idiopathic ERM [18, 44].
Collagen type IV	Promotes the fibrotic process and contributes to the formation of the hard collagen scaffold, where the myofibroblast precursor cells, when anchored there, become more susceptible to fibrogenic factors. Important component of idiopathic ERM [18, 19].
Collagen type V	Affects the mechanical properties of ERM. In addition to enhancing adhesion to various tissues, recently formed collagens may influence the rigidity and digestibility of ERM. Important component of idiopathic ERM [18, 44].
Collagen type VI	Contribution to the proliferation, migration and fibroblasts to myofibroblasts transdifferentiation, a vital procedure for mediating fibrosis, scar contraction and matrix remodeling, in many fibrotic diseases including ERM. It also forms a fine fibrillar network in idiopathic ERM. Important component of idiopathic ERM [18, 44, 83-85].

Abbreviations: ERM: epiretinal membrane

**Changes of Cytokines and Growth Factors in ERM Formation**

Cytokines and growth factors are present in both idiopathic and secondary ERMs, and in VF. These peptide factors, which are mediators of angiogenesis, may cause or aggravate diabetic neovascularization and, given that the growth and contraction of the ERM are regulated by growth factors [39], they may be implicated in idiopathic and secondary ERMs progression [1, 35, 45, 46, 86, 87]. Also, intraocular cells secrete specific cytokines and growth factors during the formation of ERM [16]. 

Cytokines contribute significantly to various inflammatory processes, to wound healing and fibrotic scarification. Fibrosis is associated with the activation of fibroblasts, which triggers collagen and glycosaminoglycan production. Release of soluble factors such as TGF-β and profibrotic mediators, like PDGF, IL-4, IL-6, IL-13 in the retina, triggers the fibrotic process via glial cells and macrophages [63, 64, 88-91]. MGCs directly participate in the formation of idiopathic and secondary ERMs by promoting fibrotic changes within the eye [63, 90]. 

Specific cytokines that participate in ERM formation and are specifically expressed in idiopathic ERMs include TGF-β [29, 92], interleukin-6 (IL-6) [29, 57], VEGF [29, 57, 92], TNF-a [25, 29], connective tissue growth factor (CTGF) [29, 93] and CCL2 [25, 29]. In contrast, there are other T-cell cytokines and associated factors, such as IFNG, RORC, IL17A and TBX21, which have no correlation with ERM formation [47]. 

In addition to cytokines, growth factors are expressed in idiopathic ERMs contributing to ERM pathogenesis. These factors include IL-6, TGF-β, nerve growth factor (NGF), CTGF, glial cell-line-derived growth factor (GDNF), PDGF, basic fibroblast growth factor (bFGF), pigment epithelium-derived factor (PEDF) and VEGF, which promote the migration and/or proliferation of a heterogeneous variety of cells [9, 16, 29, 39, 94]. Other factors and receptors expressed in idiopathic and secondary (PDR or PVR) ERM include hepatocyte growth factor (HGF), monocyte chemoattractant protein-1 (MCP-1), NF-κB, activator protein-1 (AP-1), IL-8, TNF-a, angiopoietin-2 (ANGPT2) [43] and the receptors, neurotrophin receptor p75 (p75NTR), tyrosine kinase receptors (RTKs) trkA (Tropomyosin *receptor* kinase A), trkB (Tropomyosin *receptor* kinase B) and trkC (Tropomyosin *receptor* kinase C), which are involved in neurite growth [95]. 

**The Transforming Growth Factor-β Family**

The TGF-β family, in particular, has a crucial role in idiopathic and secondary ERM formation. It is involved in normal tissue repair and development of fibrosis. TGF-β members upregulate ECM production, inhibit protease synthesis and mediate both inflammatory response and myofibroblast differentiation, while retinal Müller cells and hyalocytes produce and activate TGF-β [18]. TGF-β members, in turn, regulate how Müller cells proliferate, migrate and transdifferentiate [18, 94]. However, involvement of transdifferentiated Müller cells in the synthesis of collagen in idiopathic ERM is not clarified. Specific profibrotic cytokines can induce retinal Müller cells to transdifferentiate into a myofibroblast-like phenotype, to produce a-SMA and trigger tissue contraction [18]. 

Given that the ECM network including various collagen types, enhances the migration and proliferation of ERM cells, the TGF-β involvement in pathological ECM networks (that are responsible for idiopathic ERM) may explain its increase in the VF of patients with idiopathic ERM [94].

The TGF-β1 and TGF-β2 are upregulated in both the vitreous humor and the ERMs [18] which means that they play a significant role in idiopathic ERM pathogenesis. Expression of either TGF-β1 or TGF-β2 appears to be secondary in ERM, although there are indications that they may be involved in the origin of idiopathic ERM. TGF-β1 and NGF mRNA expression are enhanced in idiopathic ERM, while there may be differences in TGF-β1 and TGF-β2 levels within the vitreous. Indeed, the level of TGF-β2 seems to increase in the vitreous of eyes with idiopathic ERM, while the level of TGF-β1 does not increase. Previous studies of secondary (PDR and PVR) ERMs [9] indicated that TGF-β1 was the more relevant cytokine for ERM formation, detected in the vitreal humor of ERM patients and playing an essential role in fibroblast activities. However, the difference in TGF-β1 and TGF-β2 levels within the vitreous indicates that contribution of TGF-β1 in the pathogenesis of idiopathic ERM may be relatively insignificant. Moreover, presence of several activated cytokines characterizes the vitreal humor of ERM patients [63]. 

TGF-β2 is, therefore, associated with development of idiopathic and secondary ERM and its levels are correlated with intraocular fibrosis [9, 15]. Moreover, age and cell signaling seem to be associated with production of TGF-β2 in ERM [9]. The perceived prominence of TGF-𝛽2 as the most important growth factor in idiopathic ERM pathogenesis, which may stimulate the differentiation of glial cells or hyalocytes into myofibroblasts and trigger ERM contraction, makes TGF-β2 a significant therapeutic target to prevent idiopathic ERM formation and contraction [9, 15]. Despite the fact, the underlying molecular mechanism is still not fully elucidated [94]. 

**Nerve Growth Factor**

NGF can also contribute to the myofibroblast differentiation. In addition, NGF receptors may have a crucial role in this process. Hence, NGF may support the development of idiopathic ERM via the fine tuning of bound versus free NGF levels. Both increased expression and utilization of NGF may be involved in intracellular and intercellular signals leading to ERM. Therefore, both TGF-β2 and NGF contribute to idiopathic ERM pathogenesis, while NGF can induce functional changes in ERM cells and be a basis for evaluation of ERM pathology [9]. 

**Vascular Endothelial Growth Factor**

VEGF is an EC- mitogen and angiogenic inducer, which is greatly expressed in various retinal cell types in case of hypoxia [96]. It contributes to ocular cell growth and proliferation and is practically undetectable in the VF under physiological conditions [16]. VEGF is a vitreoretinal growth factor [15] that contributes to the formation of idiopathic ERM [96]. In fact, VEGF-A contributes to retinal neovascularization and secondary (PDR) ERM [97], while the VEGF*-A* *gene is overexpressed* in ERMs [15, 17]. VEGF is strongly interacting with alpha B-crystallin (CRYAB). Given that CRYAB binds to VEGF protein, it is suspected, but not confirmed, that CRYAB and VEGF are collectively involved in human diabetic retinopathy. In addition, recent reports indicate that CRYAB is upregulated in neovascularization during the formation of secondary (PDR) ERM [97].

**Insulin-like Growth Factor**

Insulin-like growth factor (IGF) apparently contributes to the progression of ERMs [9, 20, 23, 66], especially during PDR [20, 98]. The IGF system involved in the inflammation process [20, 45, 65] also stimulates ERM contraction [20, 23]. 

Furthermore, protein array analysis indicates that cytokines and growth factors in the aqueous fluid (AF) and VF of eyes with idiopathic ERM do not appear to differ significantly in their concentrations. Thus, only the homodimer form of PDGF-A is overexpressed in the VF compared to the AF [16].

**The Platelet-Derived Growth Factor Receptor**

The PDGF receptor and its subunits have been investigated in patients with secondary (PVR) ERM, whereby the PDGFRa subunit is more frequently encountered than the PDGFR𝛽 subunit [99-101]. PDGF binds to the PDGF receptor. It is an autocrine growth factor whose production is stimulated by RPE cell [99, 102]. During wound healing the interaction of PDGF with its receptor is enhanced, suggesting that PDGF may be another factor that contributes to ERM formation [99]. 

After RPE cells migration to the ERM or the vitreous cavity, they undertake the role of secreting stimulatory factors [100]. RPE cells and glial cells are attracted by PDGF, which may also function as a mitogen for these cells [103-105]. PDGF also enhance recruitment of new cells along with the proliferation of ERM cells. In addition, PDGF and VEGF take part in ERM evolution, as VEGF is localized to several secondary ERM cells [99, 105].

**Glial Cell-Line-Derived Growth Factor**

GDNF and its receptors seem to be involved in several proliferative vitreoretinal disorders, however, their role in ERM formation, especially idiopathic one, has not been widely studied. Given that GDNF levels are far below the sensitivity threshold in idiopathic ERM samples [15], there are serious doubts regarding the association of GDNF with idiopathic ERM [9]. 

**Neurotrophin Receptors**

Finally, expression of neurotrophin receptor mRNAs (trkA, trkB, trkC, and p75NTR) in idiopathic and secondary (PDR) ERMs are similar. However, NGF in idiopathic ERM is significantly higher. In contrast, GFR𝛼1 (GDNF family receptor alpha-1) receptor mRNA is higher in idiopathic ERMs compared to secondary (PDR) ERMs, while GFR𝛼2 (GDNF family receptor alpha-2) expression levels are extremely increased in secondary (PDR) ERMs [95]. 

**Changes of** **Other Proteins Specific to Idiopathic ERM**

Given the anatomically close relationship between the vitreous and the retina [106], the pathological mechanism of retinal diseases is influenced by changes in the vitreous proteins. The study of the vitreous proteome is therefore crucial to understand idiopathic ERM and differential protein expression in the AF and VF of eyes affected by idiopathic ERMs. To this date, 323 proteins have been identified in the AF and VF of patients with idiopathic ERMs [29]. Of these, 12 are differentially expressed, in a significant manner in AF versus VF; however, 8 proteins are comparably overexpressed in VF versus AF, whereas 4 proteins are similarly overexpressed in AF and VF. Fibrinogen A is the most highly expressed protein in the VF in ERM development, possibly due to local retinal inflammation caused by retinal breaks or RD. Thus, small blood vessels are dilated in this area, permitting the extravagation of soluble factors (including fibrinogen) into the VF. However, the rest of the proteins significantly overexpressed in the VF compared to the AF, or the four proteins overexpressed in the AF, are not thought to be important pathogenetic factors for idiopathic ERM [15, 16, 29].

Other studies of idiopathic ERM vitreous proteome identified (without specifically mentioning all of them) 233 proteins that are expressed at lower levels, including ESR1 protein, and 179 proteins that are expressed at higher levels. The obvious conclusion that there are severe level changes in several proteins in patients with idiopathic ERMs was also accompanied by the finding that vitreous protein concentration is, on the average, significantly higher in idiopathic ERM [29]. In idiopathic ERMs there are several proteins including cytokines and growth factors that are less abundantly expressed in contrast to others (e.g., heavy and light chains of immunoglobulin G (IgG), transferrin (TF), a1-antichymotrypsin (a1ACT), a2-HS-glycoprotein (AHSG), hemopexin (Hpx), a1- antitrypsin (A1A), serum albumin, antithrombin III (ATIII), transthyretin (TTR), apolipoprotein A-1 (Apo A-1), fibrinogen γ chain (FGG), apolipoprotein J (Apoj), haptoglobin-1 (Hp) and AP-1) that are more abundantly expressed [15, 39, 107]. It is interesting that these proteins are also common in macular holes (MHs), indicating that ERMs and MHs follow similar inflammatory processes [15]. Interestingly, matrix metalloproteinase and plasminogen are present in the idiopathic ERM vitreous proteome, while lower levels are detected for most cytoskeleton proteins, indicating that there are severe proteins changes in idiopathic ERM process [29]. 

The UBE2O (Ubiquitin-conjugating enzyme E2 O) protein is upregulated in the idiopathic ERM proteome and the vitreous. Ubiquitination is a significant enzymatic post-translational modification of all eukaryotic organisms. Post-translational modification of proteins by ubiquitin (Ub) determines several functions including DNA repair, procedures of endocytosis and cellular signaling and control of protein quality. Idiopathic ERM pathogenesis is associated with protein misfolding and subsequent aggregation. These procedures influence negatively proteins while they also decrease production of new proteins. However, validation of UBE2O as a protein biomarker of idiopathic ERMs needs to be evaluated on larger samples [29].

A long part of LR11 (11 ligand-binding repeats, also known as SorLA or SORL1) is thought to play a key role in the pathogenesis of idiopathic ERM and its VF levels of eyes with idiopathic ERM, especially of the proliferative subtype, are apparently upregulated. LR11 is considered a risk factor for the progression of idiopathic ERM given that it provokes migration and proliferation of several cells, and as its possible association with the TGF-β2 signals. LR11 is thought to act independent from clinical characteristics such as age, hypertension and diabetes mellitus [94].

Finally, removed idiopathic ERM specimens were immunostain positive for GFAP, vimentin, CD45, CD68, CD163 and cellular retinaldehyde binding protein (CRALBP), which indicates the role of hyalocytes and glial cells [15]. GFAP, in particular, is elevated in both idiopathic and secondary (PDR) ERM formation [22]. 

**Changes of Proteins That Occur in Both Idiopathic and Secondary ERMs**

Apart from proteins specific to idiopathic ERM, there is a great variety of proteins involved in both idiopathic and secondary ERMs. Proteins like FN, laminin and VTN promote cell adhesion and contribute to idiopathic and secondary (PDR) ERM formation. FN, in particular, is a multifunctional glycoprotein that its association with ERM was described more than 10 years ago [6]. It promotes cell-to-cell and cell–to-substrate adhesion and is thought to be one of the earliest factors involved in wound healing. It is also involved in the formation of transmembrane links between different cells and of temporary scaffolds, thus providing structural integrity and contraction. FN is also a key participant in cell differentiation, migration and proliferation of fibroblasts and ECs, events that are characteristic of ERM formation [6, 84].

A gene with 50 exons encodes FN mRNA. It codes extra domains A (EDA) and B (EDB), and type III homology connecting segment (IIICS), each identified by alternative splicing. Differential pre-mRNA processing potentially leads to 21 different FN isoforms. The EDB FN variant, in particular, is highly expressed in proliferating tissues (e.g., in embryos or tumors), but is not detected in normal adult tissues, the main reason it has been proposed as an angiogenic marker [6].

Ιn both idiopathic and secondary (PDR) ERMs, collagen type IV, FN and EDBFN are some ECM proteins that are overexpressed. Their overexpression is associated with elevated TGF-β and endothelin-1 (ET-1), which is more pronounced in idiopathic ERMs [6]. 

Neovascularization is considered as the first step in ERM formation. Neovascularization is a process where many angiogenic factors like apelin have a significant role [35, 45, 46, 86, 87, 108]. Apelin is upregulated in secondary (PDR) ERMs, while there is increased apelin mRNA expression in secondary ERMs. Apelin signaling is an important contributor to the formation of adventitia and in angiogenesis. It triggers EC proliferation while, in vitro, it promotes migration and tube formation. Apelin, e.g., is upregulated during tumor neovascularization and thus is considered vital for embryonic vascular development. Interestingly, apelin is not co-expressed with FN, but is co-expressed with glial-cell-specific, vascular EC and RPE cell markers. It is also involved in the differentiation of glial cells and vessels, whereas its expression is upregulated in the vascular system and vessel formation. Moreover, apelin and VEGF coordinate with each other in angiogenesis and gliosis, as they are co-expressed in secondary ERMs [35]. 

Apelin is also present in the vascular and glial components of ERMs. Thus, elevated apelin expression is associated with a characteristic microenvironment around new vessels, triggered by glial cell proliferation. Furthermore, it upregulates expression of adhesion molecules and promotes cell aggregation independently of cell expansion [35].

The expression of SP1 in secondary (PDR) ERMs has not been determined. Nevertheless, the mRNA of SPI is highly expressed in secondary ERMs, while the SP1 protein is mainly colocalized with VEGF which is significant for ERM formation. SP1-like proteins bind to GC-rich motifs of many promoters and are involved in several cellular activities. The expression of various genes like TGF-β, VEGF fibrogenic factors and matrix genes is regulated by SP1, thus these cytokines may be detected in the VF and secondary ERMs. Moreover, SP1 may be crucial for overexpression of VEGF, given its central role in angiogenesis, cancer and ERM pathogenesis [79, 80, 109]. 

ET with its 3 isoforms ET-1, ET-2, and ET-3. ET-1, is a potent vasoconstrictor molecule involved in several types of ERM. ET is recognized by ET receptors A (ETA) and B (ETB) [22]. ET-1 is a peptide produced by EC that triggers vasoconstriction when it interacts with ETA on the VSMC. It also stimulates mitogenic action on VSMC. ET-1 gene expression in EC is induced by low levels of tissue oxygen, synthesized by human RPE cells and expressed in retinal, neural, glial and vascular cells and in the optic nerve, while a breakdown of the blood-retinal barrier is suspected to increase its release [105]. 

Little is known about the ET-1 expression and its association to fibroblast proliferation in secondary, especially PDR, ERMs. There are reports about its essential role in mediating fibrosis in secondary ERMs, although it is not clear if the membrane cells produce ET-1 [22, 110-112]. 

Nonetheless, glial cells secrete ET-1 in idiopathic as well as secondary (PVR) ERMs, whereas RPE cells secrete ET-1 only in secondary (PVR) ERMs. Therefore, ET-1 may play a central role in formation and contraction of PVR ERMs, given that glial and RPE cells contribute to this process. In addition, ETA and ETB are detected in both idiopathic and secondary (PVR) ERMs [105], while ET-1 expression is elevated in secondary (PDR) ERMs. However, the role and expression of ET-1 and ETB in secondary (PDR) ERMs remains unclear compared to idiopathic ERMs [22, 105]. 

ET-1 and S100A4 are possibly upregulated in PDR ERMs, while staining techniques show that ET-1 and S100A4 are colocalized. This indicates an association between ET-1 and fibroblastic proliferation. In addition, it indicates that idiopathic and secondary ERMs are mediated by different mechanisms, especially as ET-1 seems to be a crucial factor in advanced PDR and fibroblastic transition. Therefore, ET potentially contributes to idiopathic and secondary ERM [22].

Epithelial membrane protein-2 (EMP2) is significantly expressed in many intraocular membranes. This suggests that RPE-derived cells are a crucial or predominant cell type in idiopathic and secondary (PVR) ERMs, given that EMP2 is a regulator of integrins, and is highly expressed in the RPE. EMP2 is also expressed in many secondary (PVR) ERMs, but additional research is needed to establish the association between EMP2 and secondary ERM [113]. Older studies indicated that ECM components like FN, VTN and laminin were also significant components of secondary (PVR) ERMs, which were detected in vitreous [29].

Another protein expressed in ERM is aquaporin (AQP). AQP1 has been described to be active in several cell types of the retina during both pathological and non-pathological conditions [114-119]. AQPs are integral transmembrane water channels and implicated in migration and proliferation during idiopathic and secondary (PVR) ERM formation [114, 120-127]. Indeed, AQP1 is detected at both the protein and mRNA levels, especially at the edges of membranes from idiopathic and secondary (PVR) ERMs [114]. 

ANP (Atrial natriuretic peptide) also has a crucial role for ERM formation. The ANP protein, is a natural inhibitor of angiogenesis. ANP possibly regulates the growth of secondary (PVR) ERMs and is expressed by several cells of ERM tissue, including vascular EC, RPE cells, glial cells, macrophages, vascular smooth muscle cells (VSMC) and fibroblasts. Thus, most cells that contribute to ERM formation express ANP. Because ANP is implicated in increased glial proliferation, it might contribute to secondary (PDR) ERMs formation [96, 128]. 

Periostin (POSTN) is another protein that plays a significant role mainly in secondary (PVR) ERMs. Periostin mRNA is detected in secondary ERMs, while it is low in normal retinas. It interacts with integrins αvβ1, αvβ3 and αvβ5 under conditions of tissue development and remodeling and thus enhances cell motility. POSTN is also implicated with development of several tissues and organs, including teeth, heart valves, bone and in tumor metastasis [17]. 

Periostin is found in pathologically fibrotic areas, and it may be essential for the development of PDR-ERM. It is suggested that periostin is significantly increased in the vitreous of eyes with secondary ERMs, which indicates its association with proliferation of secondary ERMs [17]. 

Finally, Snail protein may have a role in secondary (PVR) ERMs. Snail protein is primarily expressed in the nucleus and is detected via immunofluorescent staining. Several signaling pathways are involved in its regulation. Factors like Glycogen synthase kinase-3b (GSK-3b), Phosphatidylinositol 3-kinase (PI3K), Ras, Smad and MAP kinase enhance Snail regulation by TGF-β1. The expression of Snail in secondary (PVR) ERMs implicates its role in PVR pathogenesis. Moreover, Snail transcription factor has a significant role in epithelial to mesenchymal transition in human RPE cells, induced by TGF-β1, indicating that this protein may regulate the formation of secondary (PVR) ERMs [129] (Table 5). 

**Inflammation Molecules in Idiopathic and Secondary ERM**

As already mentioned, idiopathic ERM pathophysiology involves a large number of inflammation proteins, immune reactions and cytoskeleton remolding. Therefore, classical, alternative and lectin-induced pathways of complement activation, have all significant roles in the idiopathic ERM process. Five proteins including C4A (Complement C4-A), C3 (Complement C3) and complement factor B (CFB), are increased in idiopathic ERM with C4A being significantly upregulated. In addition, inflammation occurring in idiopathic ERMs may implicate the complement and coagulation cascade pathway as contributing factors [29].

**Table 5:** Proteins (Interleukins, Trophic and Transcription Factors) Involved in ERM Pathogenesis

Protein (Abbreviation)	Protein	Type of ERM Influenced
A1A	a1- antitrypsin	Idiopathic ERM [15, 39, 100]
a1ACT	a1-antichymotrypsin	Idiopathic ERM [15, 39, 100]
AHSG	a2-HS-glycoprotein	Idiopathic ERM [15, 39, 100]
ANGPT2	Angiopoietin-2	Idiopathic and secondary (PDR or PVR) ERM [43]
ANP	Atrial natriuretic peptide	Secondary (PVR) ERM [64]
AP-1	Activator protein-1	Idiopathic and secondary (PDR or PVR) ERM [43]
Apo A-1	Apolipoprotein A-1	Idiopathic ERM [15, 39, 100]
Apoj	Apolipoprotein J	Idiopathic ERM [15, 39, 100]
AQP1	Aquaporin-1	Idiopathic and secondary (PVR) ERM [103]
ATIII	Antithrombin III	Idiopathic ERM [15, 39, 100]
bFGF	Basic fibroblast growth factor	Idiopathic and secondary (PDR or PVR) ERM [15, 91]
C3	Complement C3	Idiopathic ERM [29]
C4A	Complement C4-A	Idiopathic ERM [29]
CCL2	Chemokine (C-C motif) ligand 2	Idiopathic ERM [29]
CCL26	Chemokine (C-C motif) ligand 26	ERM formation [63]
CCL27	Chemokine (C-C motif) ligand 27	ERM formation [63]
CFB	Complement factor B	Idiopathic ERM [29]
CRYAB	Alpha B crystallin	Secondary (PDR) ERM [92]
CTGF	Connective tissue growth factor	Idiopathic ERM [29, 89]
CXCL6	Chemokine (C-X-C motif) ligand 6	ERM formation [63]
CXCL10	Chemokine (C-X-C motif) ligand 10	ERM formation [63]
CXCL11	Chemokine (C-X-C motif) ligand 11	ERM formation [63]
EMP2	Epithelial membrane protein-2	Idiopathic and secondary (PVR) ERM [29]
ET	Endothelin	Idiopathic and secondary (PDR) ERM [6]
HGF	Hepatocyte growth factor	Idiopathic and secondary (PDR or PVR) ERM [43]
Hp	Haptoglobin-1	Idiopathic ERM [15, 39, 100]
FGG	Fibrinogen γ chain	Idiopathic ERM [15, 39, 100]
FN	Fibronectin	Idiopathic and secondary (PDR) ERM [6]
GDNF	Glial cell line derived growth factor	Idiopathic [9, 15] and secondary (PDR or PVR) ERM [91]
GFAP	Glial fibrillary acidic protein	Idiopathic and secondary (PDR) ERM [22]
GFR𝛼1	GDNF family receptor alpha-1	Idiopathic ERMs [91]
GFR𝛼2	GDNF family receptor alpha-2	Secondary (PDR) ERM [91]
Hpx	Hemopexin	Idiopathic ERM [15, 39, 100]
ICAM-1	Intracellular adhesion molecule 1	Idiopathic ERM and secondary ERMs [16]
IGF	Insulin-like growth factor	Secondary (PDR) ERM [20, 46]
IgG	Immunoglobulin G	Idiopathic ERM [15, 39, 100]
IL-1b	Interleukin-1b	Idiopathic and secondary ERM [63]
IL-2	Interleukin-2	Idiopathic and secondary ERM [63]
IL-4	Interleukin-4	Idiopathic and secondary ERM [63]
IL-6	Interleukin-6	Idiopathic and secondary ERM [63]
IL-8	Interleukin-8	Idiopathic and secondary (PDR or PVR) ERM [43]
IL-10	Interleukin-10	Idiopathic and secondary ERM [63]
IL-13	Interleukin-13	Idiopathic and secondary ERM [63]
MCP-1	Monocyte chemoattractant protein-1	Idiopathic and secondary (PDR or PVR) ERM [43]
NF-κB	Nuclear factor kappa-B	Idiopathic ERMs [50, 51]
NGF	Nerve growth factor	Idiopathic [9, 29] and secondary (PDR or PVR) ERM [91]
p75NTR	p75 neurotrophin receptor	Idiopathic and secondary (PDR or PVR) ERM [91]
PDGF	Platelet-derived growth factor	Idiopathic [9] and secondary ERM [65]
PDGF-A	Platelet-derived growth factor A	Idiopathic ERM [16]
PEDF	Pigment epithelium-derived factor	Idiopathic ERM [29]
POSTN	Periostin	Secondary (PVR) ERM [17]
SP1	Specificity protein 1	Secondary (PDR) ERM [79]
TF	Transferrin	Idiopathic ERM [15, 39, 100]
TGF-β	Transforming growth factor-β	Idiopathic and secondary ERM [18]
TGF-β1	Transforming growth factor-β1	Secondary (PDR and PVR) ERM [9, 18]
TGF-β2	Transforming growth factor-β2	idiopathic and secondary ERM [9, 15, 18]
TNF-a	Tumor necrosis factor alpha	Idiopathic and secondary ERM [63]
TrkA	Tropomyosin *receptor* kinase A	Idiopathic and secondary (PDR or PVR) ERM [91]
TrkB	Tropomyosin *receptor* kinase B	Idiopathic and secondary (PDR or PVR) ERM [91]
TrkC	Tropomyosin *receptor* kinase C	Idiopathic and secondary (PDR or PVR) ERM [91]
TTR	Transthyretin	Idiopathic ERM [15, 39, 100]
VEGF	Vascular endothelial growth factor	Idiopathic ERM [64]
VEGF-A	Vascular endothelial growth factor-A	Secondary (PDR) ERM [92]
VTN	Vitronectin	Idiopathic and secondary (PDR) ERM [6]
UBE2O	Ubiquitin-conjugating enzyme E2 O	Idiopathic ERM [29]

**Abbreviations: ERM: epiretinal membrane; A1A: a1- antitrypsin; a1ACT: a1-antichymotrypsin; AHSG: a2-HS-glycoprotein; ANGPT2: Angiopoietin-2; PDR: proliferative diabetic retinopathy; PVR: proliferative vitreoretinopathy; ANP: Atrial natriuretic peptide; AP-1: Activator protein-1;** **Apo A-1** **: Apolipoprotein A-1; Apoj: Apolipoprotein J; AQP1:** **Aquaporin-1** **;** **ATIII** **:** **Antithrombin III; bFGF: Basic fibroblast growth factor; C3: Complement C3; C4A: Complement C4-A; CCL2: Chemokine (C-C motif) ligand 2; CCL26: Chemokine (C-C motif) ligand 26; CCL27: Chemokine (C-C motif) ligand 27; CFB: Complement factor B; CRYAB: Alpha B crystalline; CTGF: Connective tissue growth factor; CXCL6: Chemokine (C-X-C motif) ligand 6; CXCL10: Chemokine (C-X-C motif) ligand 10; CXCL11: Chemokine (C-X-C motif) ligand 11; EMP2: Epithelial membrane protein-2; ET: Endothelin; HGF: Hepatocyte growth factor; Hp: Haptoglobin-1; FGG: Fibrinogen γ chain; FN: Fibronectin; GDNF: Glial cell line΄derived growth factor; GFAP: Glial fibrillary acidic protein; GFRα1: GDNF family receptor alpha-1; GFRα2: GDNF family receptor alpha-2 Hpx: Hemopexin; ICAM-01: Intracellular adhesion molecule 1; IGF: Insulin-like growth factor; IgG:** **Immunoglobulin G; IL-1b:** **Interleukin-1b; IL-2: Interleukin-2; IL-4: Interleukin-4; IL-6: Interleukin-6; IL-8: Interleukin-8; IL-10: Interleukin-10; IL-13: Interleukin-13; MCP-1: Monocyte chemoattractant protein-1; NF-κB: Nuclear factor kappa-B; NGF: Nerve growth factor; p75NTR: p75 neurotrophin receptor; PDGF: Platelet-derived growth factor; PDGF-A: Platelet-derived growth factor-A; PEDF: Pigment epithelium-derived factor; POSTN: Periostin; SP1: Specificity protein 1; TF: Transferrin; TGF-β: Transforming growth factor-β; TGF-β1: Transforming growth factor-β1; TGF-β2: Transforming growth factor-β2; TNF-a: Tumor necrosis factor alpha; TrkA:** **Tropomyosin *receptor* kinase A; TrkB: Tropomyosin *receptor* kinase B; TrkC: Tropomyosin *receptor* kinase C; TTR:** **Transthyretin; VEGF: Vascular endothelial growth factor; VEGF-A: Vascular endothelial growth factor-A; VTN: Vitronectin; UBE20: Ubiquitin-conjugating enzyme E2 O**

**Table 6:** Abbreviations used in the text

Expanded form	Abbreviation
a-Smooth Muscle Actin	a-SMA
Activator Protein-1	AP-1
Aquaporin	AQP
Aqueous Fluids	AF
Atrial Natriuretic Peptide	ANP
basic Fibroblast Growth Factor	bFGF
Chemokine C-X-C motif Ligand 1	CXCL1
EndoThelin-1	ET-1
EpiRetinal Membrane	ERM
Epithelial Membrane Protein-2	EMP2
ET receptors A	ETA
ET receptors B	ETB
Extra Domain A	EDA
Extra Domain B	EDB
Extracellular Matrix	ECM
Fibronectin	FN
Glial cell-line-Derived Growth Factor	GDNF
Glial Fibrillary Acidic Protein	GFAP
Glycogen Synthase Kinase-3b	GSK-3b
Hepatocyte Growth Factor	HGF
Immunoglobulin G	IgG
Insulin-like Growth Factor	IGF
Interleukin -6	IL-6
Interleukin -8	IL-8
Internal Limiting Membrane	ILM
Intracellular Adhesion Molecule 1	ICAM-1
11 Ligand-binding Repeats (also known as SorLA or SORL1)	LR11
Macular Holes	MHs
Monocyte Chemoattractant Protein-1	MCP-1
Müller Glial Cells	MGCs
Natriuretic Peptide	NP
Nerve Growth Factor	NGF
Nuclear Factor Kappa B	NF-κB
Phosphatidylinositol 3-Kinase	PI3K
Pigment Epithelium-Derived Factor	PEDF
Platelet-Derived Growth Factor	PDGF
Platelet-Derived Growth Factor A	PDGF-A
Posterior Vitreous Detachment	PVD
Preretinal Macular Fibrosis	PMF
Proliferative Diabetic Retinopathy	PDR
Proliferative Vitreoretinopathy	PVR
Retinal Detachment	RD
Retinal Pigment Epithelium cells	RPE
Tenascin C	TNC
Transforming Growth Factor	TGF
Transforming Growth Factor-β	TGF-β
Tropomyosin *Receptor* Kinase A	TrkA
Tropomyosin *Receptor* Kinase B	TrkB
Tropomyosin *Receptor* Kinase C	TrkC
Tumor Necrosis Factor alpha	TNFα
Type III homology Connecting Segment	IIICS
Tyrosine Kinase Receptors	RTKs
Ubiquitin	Ub
v-REL avian reticuloendotheliosis viral oncogene homolog A	RELA
Vascular Endothelial Growth Factor	VEGF
Vascular Endothelial Growth Factor-A	VEGF-A
Vascular Smooth Muscle Cells	VSMC
Vitreous Fluids	VF
Wild-type p53-Induced Phosphatase 1	Wip1

Cellular adhesion molecules also have an essential role for the binding of circulating inflammatory cells to specific sites, while they play a crucial role in cell proliferation. There are 25 cell adhesion proteins detected in AF and VF compartments of eyes affected by idiopathic ERM. The protein intracellular adhesion molecule 1 (ICAM-1) is detectable in the VF of eyes with idiopathic ERM, in contrast to AF from the same eyes, while it is frequently detected in the ECM of idiopathic ERM and in the endothelium of secondary ERMs [16].

Development of ERMs strongly correlates with the presence of CD44 and VCAM-1. CD44, a cell-surface glycoprotein, is involved in a wide variety of functions, including cell migration and adhesion, tumor growth and progression, also it is receptor for hyaluronic acid [17, 130]. In addition, it is involved in epithelial to mesenchymal transition, which is induced by TNF-a. VCAM-1 interacts with VLA4 and might influence immune responses and leukocyte emigration to inflammation sites [17, 131]. The apparent coordinated upregulation of CD44 and VCAM-1 found in the vitreous of eyes with secondary ERMs indicate their significant involvement in the development of secondary (PVR) ERMs [17].

Finally, development of fibrotic and inflammatory processes is related to increased levels of IL-1b, IL-2, IL- 4, IL-6, IL-10, TNF-a and INF-c [63, 89, 132-134]. Besides, presence of several proinflammatory chemokines and cytokines such as CCL26, CCL27, CXCL6, CXCL11 and INF-c that have a vital role in immunomodulation and inflammation, emphasizes the crucial role of inflammation in ERM formation [63, 135]. Table 6 summarizes abbreviations used in the text.

## CONCLUSION

As mentioned above, the main theories proposed for the pathogenesis of ERM, involve either cellular migration or cellular proliferation. The present mini review of the past decade developments tried to summarize, highlight and cross-correlate the major findings on the function of genes involved in ERM and their association with different types of cells, genes and gene expression products implicated in ERM formation. 

Although the issue of ERM pathogenesis remains poorly resolved, certain features of ERM are already apparent;

1. With the exception of TGFb2 gene, there is apparently no overlap between the genes identified so far as being involved in idiopathic ERM, PVR-ERM and PDR-ERM (Table 3). This may imply that the pathogenesis of each of these forms of ERM may be different, but they may share similar pathways of expression as well.

2. MGCs and fibroblasts or collagen cells are involved both in idiopathic ERM and PDR-ERM. However, based on our present knowledge, it is rather evident that in each case they use different sets of cytokines. 

3. The pathogenesis of secondary ERM (Tables 3,5) or its trigger(s) remains a terra incognita and an area of great research interest.

4. Growth factors, cytokines and ECM have a central role in ERM pathogenesis , as they are implicated in several functions, including cellular signal transmission and tissue modification [9].

Finally, the great number of genes involved in ERM should serve as a warning for reaching premature conclusions regarding our expectations for potential gene manipulation-based treatments of ERM. The experience, so far, from the field of oncology argues excessive reliance on genetic data for combating cancer [136-138]. Perhaps a similar argument can be made about ERM pathogenesis. Still, similar expectations can be raised about potential contributions of genetics-based therapies for its treatment.

## DISCLOSURE

Ethical issues have been completely observed by the authors. All named authors meet the International Committee of Medical Journal Editors (ICMJE) criteria for authorship of this manuscript, take responsibility for the integrity of the work as a whole, and have given final approval for the version to be published. No conflict of interest has been presented. Funding/Support: None. 
